# Fatigue-Induced Scapular Dyskinesis in Healthy Overhead Athletes

**DOI:** 10.3389/fbioe.2020.00302

**Published:** 2020-04-21

**Authors:** Matteo Zago, Adam Kawczyński, Sebastian Klich, Bogdan Pietraszewski, Manuela Galli, Nicola Lovecchio

**Affiliations:** ^1^Dipartimento di Elettronica, Informazione e Bioingegneria, Politecnico di Milano, Italy; ^2^e4Sport Laboratory, Politecnico di Milano, Italy; ^3^Faculty of Sport Science, University School of Physical Education, Wrocław, Poland; ^4^Faculty of Physical Education, University School of Physical Education, Wrocław, Poland; ^5^Department of Public Health, Experimental and Forensic Medicine, The University of Pavia, Pavia, Italy

**Keywords:** shoulder girdle, glenohumeral joint, isokinetic protocol, muscle fatigue, dyskinesis, acromion humeral distance, supraspinatus

## Abstract

Alterations of scapular kinematics affect the whole kinematic chain, potentially leading to the impingement syndrome. This is crucial in overhead sports, where athletes perform frequent and quick upper limb actions. In this manuscript, we aimed to assess the extent to which fatigue alters scapulo-thoracic and scapulo-humeral ranges of motion (RoM), as well as scapulo-humeral movement onset during different upper limb actions. Twenty-four young healthy males aged 22 ± 2 years (height: 1.82 ± 0.06 m, body mass: 78.0 ± 7.8 kg) performed three movements (upper limb elevation, scapular-plane abduction, and intra-extra rotation) before and after an isokinetic fatigue protocol (upper limb intra/extra rotation, 32 repetitions at 120 degrees/s). Pre vs. post fatigue RoM of humeral elevation and rotation, scapular retraction/protraction, and rotation and tilt were computed. Humerus-scapula movement delay was also determined. Humerus elevation range reduced during intra/extra humerus rotation in fatigued conditions (*p* = 0.006). Scapular tilt RoM increased after the fatigue protocol (*p* = 0.063, large effect). Humerus-scapular movement onset delay reduced in fatigued conditions of about 80 ms (*p* < 0.001, large effect). In sum, fatigued intra/extra upper limb rotators altered the scapulohumeral rhythm, and joints RoM in movements outside the scapular plane. Rather, movements close to the scapular plane were less prone to fatigue-induced alterations.

## Introduction

Shoulder girdle kinematics involves the synergic motion of three bones (humerus, clavicle, and scapula) and the interaction of three joints (glenohumeral, sternoclavicular, and acromioclavicular), combined with a so called functional joint (scapulo-thoracic), which describes the rotation and sliding of the scapula on the thorax (Neumann, 2010; Lefèvre-Colau et al., 2018a, b). Alterations of this fine scapular mechanism are referred to as dyskinesis (Kibler et al., 2013; Noguchi et al., 2013) and have detrimental effects on the whole kinematic chain (Maenhout et al., 2015; Chopp-Hurley et al., 2016; Lefèvre-Colau et al., 2018b). While performing athletic actions, especially in disciplines involving repeated overhead maneuvers, a premature or excessive rotation of the scapula during humerus elevation or lowering can induce abnormal sliding of the humeral head. This in turn might lead to the impingement syndrome (Su et al., 2004; Chopp et al., 2011; Noguchi et al., 2013; Chopp-Hurley et al., 2016).

In healthy athletes, fatigue is known as a factor increasing the risk of scapular dyskinesis. The reason is that fatigue induces weakness in rotators cuff muscles [supraspinatus, infraspinatus, subscapularis, and teres minor (Chopp-Hurley et al., 2016)] and in scapula stabilizers [upper-middle-lower trapezius, serratus anterior, and latissimus dorsi (Noguchi et al., 2013)]. Further, fatigued extra-rotators might not be able to compensate the superior destabilizing shear forces exerted by the deltoid, leading to a compression of the humeral head in the glenoid cavity (Weiner and Macnab, 1970). As such, a general consensus exists on the effect of muscular fatigue on scapulo-thoracic rotations. However, their range and direction is still debated (Noguchi et al., 2013). Chopp-Hurley et al. (2016) observed a larger upward scapular rotation and unchanged tilt angle, while Noguchi et al. (2013) did not find significant changes in scapular rotation after a fatigue protocol for scapular stabilizers. Other Authors reported a range of apparently contrasting results concerning the increment or decrement of scapular rotations in various athletes populations (Su et al., 2004; Ebaugh et al., 2006; Maenhout et al., 2015). Discrepancies between studies can be attributed to the degree of humeral elevation and the postural arrangement adopted in the fatigue protocols (prone, sitting or standing position), the applied load (bottle, elastic band, and sport-specific actions) or upper limb movement (elevation or abduction) (Lawrence et al., 2019).

A natural movement shared by many sports, the humerus abduction on the scapular plane [30-degrees with respect to the coronal plane (Neumann, 2010)] can highlight changes in the scapulo-humeral rhythm. The scapulo-humeral rhythm is conventionally set at 1:2, according to the Inman’s classic ratio between the angles of the humerus and scapula, respectively (Freedman and Munro, 1966; Bagg and Forrest, 1988; Mandalidis et al., 1999; Neumann, 2010). Whether or not fatigue could alter this ratio is still an unclear subject that merits further investigation.

Therefore, this study aimed at addressing whether a close-to-exhaustion exercise involving intra and extra upper limb rotators could induce scapular dyskinesis in overhead athletes. The topic is relevant for the athletes’ health, as we hypothesize that an increased scapular rotation can be accompanied by an unchanged humerus range of motion, thus impacting on the scapulo-humeral rhythm and leading to higher impingement risk; the related timings could also be affected. In addition, angular ranges could be altered during elevation (typical of handball and volleyball), scapula abduction in the scapular plane (a natural action) or abduction on the pure coronal plane (typical of gymnastics and throwing). A better knowledge of these mechanisms could help coaches and clinicians in choosing the exercise load while minimizing the likelihood of scapular dyskinesis. Further, with a better understanding of the kinematic effects of fatigued intra and extra rotator muscles we might be able to suggest specific training strategies to prevent kinesio-pathological movement patterns.

## Materials and Methods

### Participants

A cohort of 24 healthy male participants [age: 22 (SD 2) years, stature: 1.82 (SD 0.06) m, body mass: 78.0 (SD 7.8) kg] were recruited on a voluntary basis. Each subject was training at least three times a week, 2 h per session. However, they all refrained from any intense physical activity in the 2 days preceding the test. They all provided formal written consent, after being informed on the related risks and benefits of the research. All participants were right-handed and had 5 to 10 years of training experience in disciplines involving overhead actions. Participants did not experience any history of pain or injuries at the shoulder girdle nor in the thorax/scapular region in the year before the study.

This research was approved by the Institutional Ethics Committee (protocol 26/2016) and met the current ethical standards in Exercise Research stated in the Declaration of Helsinki.

### Study Design and Procedures

This observational case-series study involved two repeated kinematic measurements taken before and following the fatigue protocol, as detailed in Klich et al. (2019). Each measurement included three upper limb movements on the right side (dominant for all subjects): (i) elevation, (ii) abduction in the scapular plane, (iii) intra-extra rotation (with the elbow flexed at 90 degrees). Participants were acquainted with the exercise in the days preceding the laboratory session, and followed the verbal and visual instructions of an experienced physiotherapist during the test. Each subject performed the actions at his personal pacing, as we were interested in measuring movement timings. A 30-s kinematic calibration procedure was also required to ensure proper joints kinematic reconstruction, and it was performed before the first and after the second test repetition, as explained in section “Data Processing.”

**FIGURE 1:**
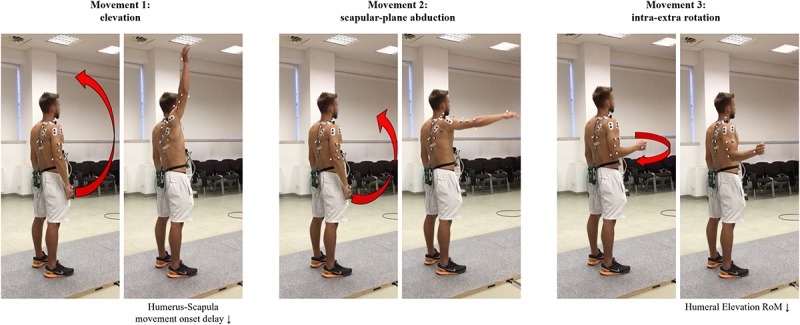
Marker placement and schematic representation of the three performed actions (initial and ending posture). Significant post-fatigue changes were also briefly reported below.

Reflective markers were placed in the following anatomical positions, according to ISB recommendations (Wu et al., 2005): C7, T8, suprasternal notch, processus xiphoideus (thorax); trigonum spinae, angulus acromialis (scapula); medial and lateral humeral epicondyles (humerus). Two additional marker clusters were placed on the posterior-lateral acromion (Andel et al., 2009) and on the upper arm for the tracking of scapular motion and for the determination of the glenohumeral joint center, respectively. The marker set is visible in Figure 1.

Between the repetitions of the kinematic tests, participants performed a fatigue protocol on an isokinetic dynamometer (Biodex Multi-Joint System 4 Pro, Biodex Medical Systems, Inc., Shirley, NY, United States). The device was set for upper limb intra-extra rotation from 0 degrees (internal rotation) to 90 degrees (external rotation), while the elbow was flexed at 90 degrees and the shoulder abducted at 90 degrees (Klich et al., 2019). Participants seated at the isokinetic dynamometer with their back against a chair and were secured with belts in order to avoid trunk or shoulder movements. The fatigue protocol consisted of three sets of 32 repetitions at isokinetic speed of 120 degrees/s, with one-minute recovery between sets, as detailed by Mullaney and McHugh (2006). Post-fatigue testing in principle, should have been done immediately upon completing the protocol, but experimental procedures delayed the second measurement up to 90 s from the end of the isokinetic protocol. Keeping rest time below 120 s still allowed the second measurement session to be conducted in real fatigue conditions (Ebaugh et al., 2006): a longer rest time would have produced a restoration of the alterations produced by the exercise at cellular (metabolic changes and mitochondrial disturbances) and blood-flow level (Cagnie et al., 2012; Rooney et al., 2014).

### Data Processing

**FIGURE 2:**
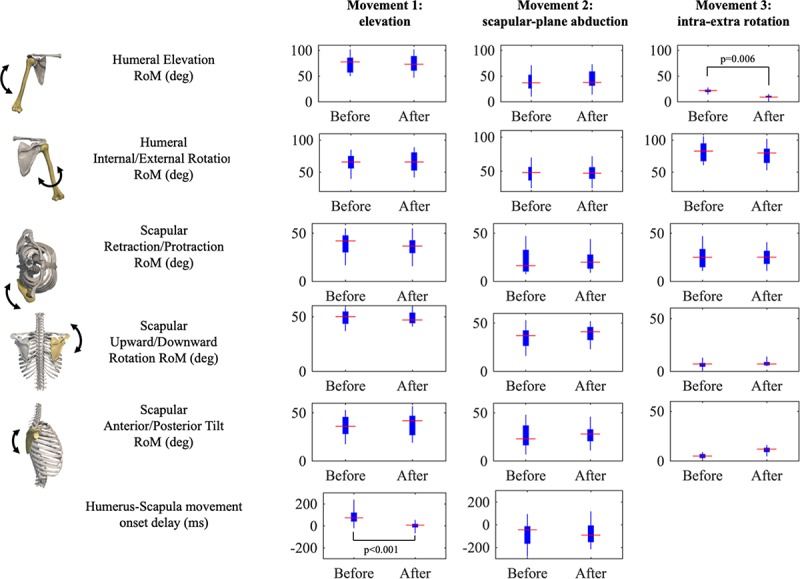
Results of joints Ranges of Motion (RoM) and Humerus-Scapula movement onset delay presented as boxplots for each movement (rows). Significant *post hoc* tests are reported.

Custom MATLAB (v. 2018b, Mathworks Inc., Natwick, United States) routines were developed. Raw three-dimensional coordinates were filtered at 6 Hz (Butterworth zero-lag, second-order low-pass filter). The glenohumeral joint center of rotation was determined through the calibration recording using the least-squares algorithm described in Gamage and Lasenby (2002). Subsequently, for the humerus and trunk we implemented the coordinate systems defined by the ISB recommendations (Wu et al., 2005), while the scapular coordinate system was slightly modified due to the marker cluster (Nicholson et al., 2014; Russo et al., 2014; Rapp et al., 2017). Scapula-thoracic angles were obtained with an YXZ Euler sequence (Wu et al., 2005), upward rotation of the scapula was defined as the inferior border of the scapula rotating laterally. Posterior tilting of the scapula was defined as the inferior border of the scapula tilting anteriorly. Scapular Retraction was defined as the medial border of the scapula moves toward the vertebral column. The scapula-humeral angles were computed as an YXY Euler sequence (Wu et al., 2005), and we considered humeral elevation and intra-extra rotation (Figures 1, 2).

**FIGURE 3:**
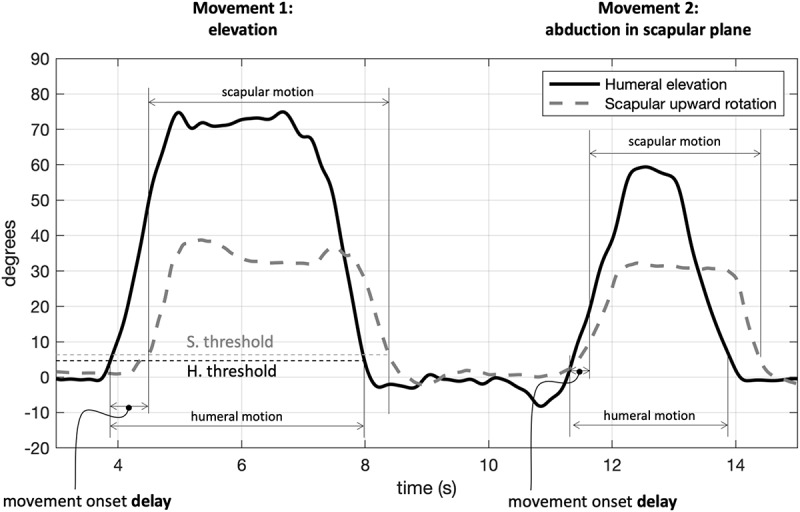
Determination of movement onset in the upper limb elevation (movement 1) and abduction in scapular plane (movement 2): movement started when exceeding the threshold by 5 degrees (+10% standard deviation) of the rest value. Data refer to a single test repetition. H, humeral; S, scapular.

Angular ranges of motion (RoM) were computed during motion. To determine the movement onset delay, we automatically set events on the time series of scapular rotation and humerus elevation: movement started when the angular value differed by more than 5 degrees + 10% SD with respect to the mean value computed in a one-second recording at rest; similarly, movement ended when the angle returned below the same threshold. Humerus-scapular movement onset delay was also computed relative to the first and second movement as the difference between the first time instant of scapular rotation and of the humerus elevation (Figure 3).

### Statistical Analysis

After checking for normality with Jarque-Beta tests, we tested condition-related differences in the RoM and delay variables. A repeated-measures ANOVA design was implemented with factors Condition (before and after the fatigue protocol) and Movement. For readability, ANOVA results were limited to the Condition factor and the interaction between Condition and Movement. *Post hoc* tests were performed adopting a Dunn-Sidak correction of the significant threshold. The alpha level was set at 0.05. Cohen’s *d* Effect Size was computed for paired *post hoc* comparisons. Effects < 0.3, between 0.3 and 0.5 and larger than 0.8 were considered small, medium and large, respectively (Cohen, 1992).

## Results

All RoMs were comparable from before to after the fatigue protocol (*p* > 0.05, low-to-medium effects), with the exception of humerus elevation in the third movement (internal/external humerus rotation), which was reduced in fatigued conditions (*p* = 0.006), as reported in Table 1. A tendency to increase in RoM scapular tilt was observed after the fatigue protocol (large effect, *p* = 0.063). As expected, we found substantial differences in joints RoM across the three movements (Movement factor, *p* < 0.001). Humerus-scapular movement onset delay reduced in fatigued conditions of about 80 ms (*p* < 0.001, large effect), with a high interindividual variability and a significant Condition × Movement interaction.

**TABLE 1:** Mean (SD) of angular ranges of motion (deg) of Humerus (H), Scapula (S), and humerus-scapula movement onset delay; ANOVA’s Condition factor and Condition × Movement interaction results are reported.

	**Movement 1:** **Elevation**			**Movement 2:** **Scapular-plane abduction**			**Movement 3:** **Intra-extra rotation**			**Condition**		**Condition ×** **Movement**	
**Variable**	**Pre**	**Post**	***d***	**Pre**	**Post**	***d***	**Pre**	**Post**	***d***	***F***	***p***	***F***	***p***
H. Elevation	72.9(16.9)	73.1(18.5)	0.02	39.8(18.5)	42.6(18.6)	0.15	21.3(3.9)	9.3(3.2)	1.71	3.34	**0.038**	1.45	0.230
H. Rotation	65.4(12.5)	65.3(14.9)	0.00	47.1(14.2)	47.1(11.8)	0.00	82.5(14.6)	76.5(15.0)	0.40	0.69	0.499	0.71	0.400
S. Retraction	39.5(11.2)	35.9(10.8)	0.33	21.3(12.8)	22.0(10.1)	0.07	25.7(11.2)	25.2(8.6)	0.06	0.49	0.611	0.34	0.531
S. Rotation	48.9(6.5)	48.2(5.6)	0.12	35.1(10.3)	39.7(8.4)	0.47	6.4(2.8)	7.6(2.6)	0.42	1.87	0.158	2.12	0.148
S. Tilt	36.5(10.3)	37.8(11.8)	0.11	25.9(11.9)	27.4(9.4)	0.14	5.1(2.3)	10.9(3.2)	1.45	0.94	0.394	3.39	0.068
Delay (ms)	84.29(59.26)	1.12(34.43)	1.31	−80.39(105.59)	−75.04(97.34)	0.05	−	−	−	6.76	**0.011**	5.23	**0.025**

*D, Cohen’s *d* effect size, significant *p* values in bold.*

## Discussion

The main finding of this study is that fatigue altered the timing of the scapulo-humeral rhythm, while scapula-humeral and scapula-thoracic ranges of motions were only slightly affected. The understanding of the mechanisms leading to the impingement syndrome and humeral head migration is the first step toward an effective prevention plan in overhead athletes. This in turn leads to great benefits in terms of sport practice, clinical care and social burden and most important, pain reduction and quality of life improvements.

### Fatigue-Induced Kinematical Alterations

In this study, we investigated the changes in the humeral and scapular rotation following an objective fatigue protocol (isokinetic exercise) on the intra and extra rotator muscles (Klich et al., 2019). Weakness in such muscle groups were previously associated to variations in scapula-humeral rotations (dyskinesis) and to the migration of the humeral head toward the acromion (Maenhout et al., 2015; Chopp-Hurley et al., 2016).

We did not find any significant change in the range of humeral rotations in the scapular-plane abduction (first movement); at the scapular level, just slight (and not-significant) variations were observed, as in Maenhout et al. (2015). This result should be considered together with the other minor changes in rotations (see Figure 2), which substantially did not affect the timing of humerus and scapula (non-significant movement onset delay). Conversely, scapular upward rotation range slightly tended to increase in the second and third movements (not significantly but with a medium effect size). We hypothesize this could be a sort of conservative adaptation to prevent impingement. This finding is in line with those by Maenhout et al. (2015), who proposed a similar fatigue protocol. Also (Chopp-Hurley et al., 2016) found changes in the upward scapula rotation. Interestingly (Noguchi et al., 2013), did not find any modification in rotation, tilt and retraction after a fatigue protocol focused on stabilizers as trapezius, serratus, and latissimus dorsi. Since we observed a dyskinesia in fatigued conditions (i.e., retraction reduction), which could in turn deteriorate the scapulo-humeral rhythm, we can argue fatigued extra rotators might be the responsible of both dyskinesis and changes in the scapular rotation and tilt (Ebaugh et al., 2006).

The significant reduction of humeral elevation RoM in fatigued conditions suggests a strategy by which the humerus tends to reduce its excursion to avoid upward translation. This constitutes an example of preventive movement pattern operated by the neuromuscular controller to preserve its own integrity (Sahrmann, 2014). While not significant, scapular anterior tilt almost doubled during intra-extra upper limb rotations (large effect), and upward/downward rotation increased with medium effect. These findings suggest a pattern where the increased scapular motion compensated for the reduced humeral rotation (large effect) induced by fatigue. In addition, Ebaugh et al. (2006) observed clearer variations in scapular rotations corresponding to a humerus elevation between 60 and 90 degrees, with a reduction in posterior tilt. Maenhout et al. (2015) measured an increased scapular rotation in all the three planes in overhead athletes following a fatigue protocol based on throwing actions. However, absolute ranges measured in the current study were aligned with those of Chopp-Hurley et al. (2016) and Ebaugh et al. (2006).

Therefore, in the light of our and previously published results (Ebaugh et al., 2006; Chopp et al., 2011; Noguchi et al., 2013; Chopp-Hurley et al., 2016), we could argue that fatigue-induced dyskinesis does not manifest itself as a defined change in a unique parameter in all individuals. Rather, fatigue can produce a sequence of minor alterations (sometimes opposed or inconsistent) that would not be relevant alone, but that can lead to disfunction when considered together. In these terms, preventing rotator muscles weakness and adopting scapular-plane actions can be both effective strategies to preserve athletes’ integrity. It also appeared that upper limb movements closer to the trunk would ease the action of the stabilizers and of the serratus anterior, responsible of scapular protraction (Neumann, 2010), also facilitating the eccentrical contraction of the middle trapezius and rhomboid major. These muscle groups control the scapular protraction.

### Limitations

The assessed cohort involved overhead athletes from different sports and adopted a quantitative yet general isokinetic fatigue protocol: as such, the present work shares with previous investigations the non-specificity of the fatigue protocol (Ebaugh et al., 2006; Chopp et al., 2011; Noguchi et al., 2013) and the general characteristics of the studied cohort (Tyler et al., 2009; Maenhout et al., 2015). A fatigue exercise tailored on discipline-specific actions would enhance the ecological validity of the results (as athletes used to fatigue in their sport could have already some adaptations to fatigue) and possibly highlight further insights (Edmonds and Dengerink, 2014; Zago et al., 2019).

## Conclusion

Fatigued intra and extra upper limb rotators altered the scapulohumeral rhythm and to some extent joints RoM when movement were out of the scapular plane. Our findings suggest that in fatigued conditions we should place particular attention in requesting athletes to perform this kind of actions and possibly to avoid elevations of more than 90 degrees (Chopp et al., 2011; Maenhout et al., 2015; Chopp-Hurley et al., 2016). Rather, athletes should be instructed to perform as much as possible movements close to the scapular plane, which resulted in less fatigue-induced kinematic alterations.

In this terms, eccentric strength training focused on middle trapezium and rhomboids might also help as a prevention strategy. Additionally, intra-rotators muscle are the primary movement drivers in explosive overhead actions (e.g., throw, pitch, and spike, etc.), thus they are more likely to get fatigued earlier. An increase in muscle tone of these groups might prevent negative scapular tilt alterations preserving a correct glenoid orientation. In conclusion, it is worth noticing that given the multifactorial (and in principle discipline-specific) nature of fatigue-induced kinematic effects, these results do not allow to devise a single, a universal exercise/program able to prevent dyskinesis. Providing adequate between- and within-sessions rest periods appears the only reliable approach to minimize the risk of such impairment.

## Data Availability Statement

The datasets generated for this study are available on request to the corresponding author.

## Ethics Statement

Written informed consent was obtained for the publication of any identifiable images.

## Author Contributions

AK and NL constructed the strains. MZ planned the study. MZ, SK, BP, and NL carried out all phases of the protocol. MG supervised the kinematic and isokinetic settings. MG, SK, and MZ performed the statistical analysis. MZ and NL drafted the manuscript. All authors read and approved the final version of the manuscript.

## Conflict of Interest

The authors declare that the research was conducted in the absence of any commercial or financial relationships that could be construed as a potential conflict of interest.

## References

[B1] Andel C. Van, Hutten K. Van, Eversdijk M. Veeger D. (2009). Gait & posture recording scapular motion using an acromion marker cluster. *Gait Posture* 29 123–128. 10.1016/j.gaitpost.2008.07.012 18815043

[B2] Bagg S. D. Forrest W. J. (1988). A biomechanical analysis of scapular rotation during arm abduction in the scapular plane. *Am. J. Phys. Med. Rehabil.* 67 238–245. 10.1097/JSM.0b013e31822179e8 3196449

[B3] Cagnie B. Barbe T. De Ridder E. Van Oosterwijck J. Cools A. Danneels L. (2012). The influence of dry needling of the trapezius muscle on muscle blood flow and oxygenation. *J. Manipul. Physiol. Ther.* 35 685–691. 10.1016/j.jmpt.2012.10.005 23206963

[B4] Chopp J. N. Fischer S. L. Dickerson C. R. (2011). The specificity of fatiguing protocols affects scapular orientation: implications for subacromial impingement. *Clin. Biomech.* 26 40–45. 10.1016/j.clinbiomech.2010.09.001 20926168

[B5] Chopp-Hurley J. N. O’Neill J. M. McDonald A. C. Maciukiewicz J. M. Dickerson C. R. (2016). Fatigue-induced glenohumeral and scapulothoracic kinematic variability: implications for subacromial space reduction. *J. Electromyogr. Kinesiol.* 29 55–63. 10.1016/j.jelekin.2015.08.001 26320811

[B6] Cohen J. (1992). A power primer. *Psychol. Bullettin* 112 155–159. 10.1037//0033-2909.112.1.155 19565683

[B7] Ebaugh D. D. McClure P. W. Karduna A. R. (2006). Scapulothoracic and glenohumeral kinematics following an external rotation fatigue protocol. *J. Orthop. Sport. Phys. Ther.* 36 557–571. 10.2519/jospt.2006.2189 16915977

[B8] Edmonds E. W. Dengerink D. D. (2014). Common conditions in the overhead athlete. *Am. Fam. Phys.* 89 537–541. 24695599

[B9] Freedman L. Munro R. R. (1966). Abduction of the arm in the scapular plane: scapular and glenohumeral movements. A roentgenographic study. *J. Bone Joint Surg. Am.* 48 1503–1510. 10.2106/00004623-196648080-00004 5955639

[B10] Gamage S. S. H. U. Lasenby J. (2002). New least squares solutions for estimating the average centre of rotation and the axis of rotation. *J. Biomech.* 35 87–93. 10.1016/S0021-9290(01)00160-9 11747887

[B11] Kibler W. Ben, Ludewig P. M. McClure P. W. Michener L. A. Bak K. (2013). Clinical implications of scapular dyskinesis in shoulder injury: the 2013 consensus statement from the “scapular summit. *Br. J. Sports Med.* 47 877–885. 10.1136/bjsports-2013-092425 23580420

[B12] Klich S. Pietraszewski B. Zago M. Galli M. Lovecchio N. Kawczyñski A. (2019). Ultrasonographic and myotonometric evaluation of the shoulder girdle after an isokinetic muscle fatigue protocol. *J. Sport Rehabil.* 10.1123/jsr.2019-0117 [Epub ahead of print]. 31593927

[B13] Lawrence R. L. Braman J. P. Ludewig P. M. (2019). The impact of decreased scapulothoracic upward rotation on subacromial proximities. *J. Orthop. Sport. Phys. Ther.* 18 1–40. 10.2519/jospt.2019.8590 PMC7112160 30658048

[B14] Lefèvre-Colau M. M. Nguyen C. Palazzo C. Srour F. Paris G. Vuillemin V. (2018a). Kinematic patterns in normal and degenerative shoulders. Part II: review of 3-D scapular kinematic patterns in patients with shoulder pain, and clinical implications. *Ann. Phys. Rehabil. Med.* 61 46–53. 10.1016/j.rehab.2017.09.002 28987866

[B15] Lefèvre-Colau M. M. Nguyen C. Palazzo C. Srour F. Paris G. Vuillemin V. (2018b). Recent advances in kinematics of the shoulder complex in healthy people. *Ann. Phys. Rehabil. Med.* 61 56–59. 10.1016/j.rehab.2017.09.001 28964876

[B16] Maenhout A. Dhooge F. Van Herzeele M. Palmans T. Ann C. (2015). Acromiohumeral distance and 3-dimensional scapular position change after overhead muscle fatigue. *J. Athl. Train.* 50 281–288. 10.4085/1062-6050-49.3.92 25594913 PMC4477924

[B17] Mandalidis D. G. Mc Glone B. S. Quigley R. F. McInerney D. O’Brien M. (1999). Digital fluoroscopic assessment of the scapulohumeral rhythm. *Surg. Radiol. Anat.* 21 241–246. 10.1007/s00276-999-0241-4 10549079

[B18] Mullaney M. McHugh M. (2006). Concentric and eccentric muscle fatigue of the shoulder rotators. *Int. J. Sports Med.* 57 725–729. 10.1055/s-2005-872870 16586324

[B19] Neumann D. A. (2010). *Kinesiology of the Musculoskeletal System: Foundations for Rehabilitation.* St. Luois: Mosby, 10.1029/2007JG000640/abstract

[B20] Nicholson K. F. Russo S. A. Kozin S. H. Zlotolow D. A. Hulbert R. L. Rowley K. M. (2014). Evaluating the acromion marker cluster as a method for measuring scapular orientation in children with brachial plexus birth palsy. *J. Appl. Biomech.* 30 128–133. 10.1123/jab.2012-0180 24676519

[B21] Noguchi M. Chopp J. N. Borgs S. P. Dickerson C. R. (2013). Scapular orientation following repetitive prone rowing: implications for potential subacromial impingement mechanisms. *J. Electromyogr. Kinesiol.* 23 1356–1361. 10.1016/j.jelekin.2013.08.007 24055533

[B22] Rapp E. A. Richardson R. T. Russo S. A. Rose W. C. Richards J. G. (2017). Short communication A comparison of two non-invasive methods for measuring scapular orientation in functional positions. *J. Biomech.* 61 269–274. 10.1016/j.jbiomech.2017.07.032 28823505

[B23] Rooney S. I. Loro E. Sarver J. J. Peltz C. D. Hast M. W. Tseng W. J. (2014). Exercise protocol induces muscle, tendon, and bone adaptations in the rat shoulder. *Muscles Ligaments Tendons J.* 4 413–419. 10.11138/mltj/2014.4.4.413 25767777 PMC4327349

[B24] Russo S. A. Kozin S. H. Zlotolow D. A. Thomas K. F. Hulbert R. L. Mattson J. M. (2014). Scapulothoracic and glenohumeral contributions to motion in children with brachial plexus birth palsy. *J. Shoulder Elb. Surg.* 23 327–338. 10.1016/j.jse.2013.06.023 24075782

[B25] Sahrmann S. A. (2014). The human movement system: our professional identity. *Phys. Ther.* 12 862–869. 10.2522/ptj.20130319 24627430

[B26] Su K. P. E. Johnson M. P. Gracely E. J. Karduna A. R. (2004). Scapular rotation in swimmers with and without impingement syndrome: practice effects. *Med. Sci. Sports Exerc.* 36 1117–1123. 10.1249/01.MSS.0000131955.55786.1A 15235314

[B27] Tyler T. F. Cuoco A. Schachter A. K. Thomas G. C. McHugh M. P. (2009). The effect of scapular-retractor fatigue on external and internal rotation in patients with internal impingement. *J. Sport Rehabil.* 18 229–239. 10.1123/jsr.18.2.229 19561366

[B28] Weiner D. S. Macnab I. (1970). Superior migration of the humeral head. A radiological aid in the diagnosis of tears of the rotator cuff. *J. Bone Joint Surg. Br.* 52 524–527. 10.1302/0301-620x.52b3.524 5455085

[B29] Wu G. Helm F. C. T. Van Der, Veeger H. E. J. D. Makhsous M. Roy P. (2005). ISB recommendation on definitions of joint coordinate systems of various joints for the reporting of human joint motion — Part II: shoulder, elbow, wrist and hand. *J Biomech.* 38 981–992. 10.1016/j.jbiomech.2004.05.042 15844264

[B30] Zago M. Esposito F. Bertozzi F. Tritto B. Rampichini S. Galvani C. (2019). Kinematic effects of repeated turns while running. *Eur. J. Sport Sci.* 19 1072–1081. 10.1080/17461391.2019.1578416 30836850

